# IRF4 suppresses osteogenic differentiation of BM-MSCs by transcriptionally activating miR-636/DOCK9 axis

**DOI:** 10.1016/j.clinsp.2022.100019

**Published:** 2022-04-06

**Authors:** Xuepu Zhang, Yue Zhang, Limin Yang, Yuexin Wu, Xiaohu Ma, Gang Tong, Zhaoliang Ban, Haosen Zhao

**Affiliations:** a Orthopedics, The First Affiliated Hospital of Jinzhou Medical University, China; b Dental Department, The Second Affiliated Hospital of Jinzhou Medical University, China

**Keywords:** Osteoporosis, BM-MSCs, Osteogenic differentiation, miR-636, DOCK9, ALP

## Abstract

**Objectives:**

Osteoblasts are derived from Bone Marrow-derived Mesenchymal Stem Cells (BM-MSCs), which play an indispensable role in bone formation. In this study, the authors aim to investigate the role of IRF4 in the osteogenic differentiation of BM-MSCs and its potential molecular mechanism.

**Methods:**

The authors used lentivirus infection to overexpress IRF4 in BM-MSCs. The expression of IRF4 and osteogenesis-related genes were detected by qRT-PCR and western blot analysis. The osteogenic differentiation of BM-MSCs was evaluated by Alkaline Phosphatase (ALP) activity, Alizarin red staining, and Alkaline Phosphatase (ALP) staining. Chromatin Immunoprecipitation (ChIP), Dual-Luciferase reporter assay and RNA Immunoprecipitation Assay were applied to confirm the regulatory mechanism between IRF4, miR-636 and DOCK9.

**Results:**

The authors found IRF4 was down-regulated during the osteogenic differentiation of BM-MSCs, and IRF4 overexpression could decrease the osteogenic differentiation of BM-MSCs by specifically promoting the reduction of Alkaline Phosphatase (ALP) activity and down-regulating osteogenic indicators, including OCN, OPN, Runx2 and CollA1. Mechanistically, IRF4 activated microRNA-636 (miR-636) expression via binding to its promoter region, and Dedicator of Cytokinesis 9 (DOCK9) was identified as the target of miR-636 in BM-MSCs. Moreover, the damage in the capacity of osteogenic differentiation of BM-MSCs induced by IRF4 overexpression could be rescued by miR-636 inhibition.

**Conclusions:**

In summary, this paper proposed that IRF4/miR-636/DOCK9 may be considered as targets for the treatment of osteoporosis (OP).

## Highlights

• IRF4 is a negative regulator during the osteogenic differentiation of BM-MSCs.
• IRF4 can transcriptionally activate microRNA-636 in BM-MSCs.
• MicroRNA-636 can suppress the osteogenic differentiationof BM-MSCs by directly targeting DOCK9.

## Introduction

Osteoporosis is a metabolic bone disease caused by loss of bone mass, destruction of bone microstructure, and increase of bone fragility.^1^ Osteoporosis is mainly classified into primary or secondary osteoporosis. Primary osteoporosis commonly occurs in menopause women and the elder, which could further be dived into postmenopausal osteoporosis, idiopathic osteoporosis, and age-related osteoporosis. Secondary osteoporosis is resulted from medications, other conditions or diseases.^2^ Activated osteoclasts and reduced osteoblasts can drive bone absorption, resulting in decreased bone mass and inducing osteoporosis.^3^^,^^4^ Therefore, the identification of biological targets involved in the proliferation and differentiation of osteoblasts is critical to ameliorating osteoporosis.

Interferon Regulatory Factor 4 (IRF4) is a subset of transcription factors of the Interferon Regulatory Factor (IRF) family. Previous reports mainly focus on the roles of IRF4 in immune infiltration,^5^^,^^6^ macrophage polarization,^7^ cell cycle^8^ and proliferation.^9^ IRF4 has been proved to be involved in the onset and progression of multiple diseases, such as cancers and stroke.^10^, ^11^, ^12^, ^13^ For instance, IRF4 mediated by the hsa_circ_0000301/hsa-miR-1228-3p axis may be involved in the occurrence and development of cervical cancer.^10^ Chang et al. revealed that IRF4 demethylated by H3K27 demethylase KDM6B was related to ischemic brain injury.^11^ Lei and his colleagues disclosed that upregulation of the genes in the IRF family, including IRF1, IRF2, IRF3, IRF4, IRF5, IRF7, IRF8, and IRF9, was highly correlated with the poor prognosis of human glioma.^12^ Few studies has reported the influence of IRF4 in osteoporosis the authors have noticed Nakashima et al. revealed that IRF4 was highly expressed in RANKL-activated osteoclasts, and accelerated osteoclast differentiation through binding to the promoter region of NFATc1.^13^ The onset of osteoporosis results from the alteration of the homeostatic balance, especially the activation of osteoblasts and the inactivation of osteoclasts. So, the authors wonder whether IRF4 is also involved in osteogenic differentiation.

MicroRNA-636 (miR-636) is a member of microRNAs (miRNAs), and miR-636 plays critical roles in various diseases, such as endometrial carcinoma,^14^ prostate cancer,^15^ and atherosclerosis.^16^ In addition, miR-636 was involved in pulmonary inflammation in cystic fibrosis.^17^ Up to date, the role of miR-636 in the onset and development of osteoporosis remains elusive. Few studies have reported the influence of DOCK9 in osteoporosis

Dedicator of Cytokinesis 9 (DOCK9) belongs to the Dedicator of Cytokinesis D (DOCK-D) family, which is classified into 11 DOCK proteins from DOCK1 to DOCK11.^18^ According to previous evidence, DOCK is closely associated with irregular astigmatism and corneal ectasia.^19^ In addition, it has been demonstrated that DOCK proteins modulate actin cytoskeleton formation, cell adhesion, and migration.^20^ However, whether DOCK9 is related to osteogenic differentiation remains unknown.

In this study, it was hypothesized that IRF4 might transcriptionally activate miR-636, and the relationship between IRF4 and miR-636 during osteogenic differentiation was determined. Furthermore, the downstream target genes of miR-636 were identified, and the effect of IRF4/miR-636/DOCK9 on osteogenic differentiation was investigated for the treatment of osteoporosis.

## Materials and methods

### Cell culture and treatment

Human Bone Marrow Mesenchymal Stem Cells (BM-MSCs) were supplied by Saliai Stem Cell Science and Technology (Guangzhou) and were cultured in low-glucose 'Dulbecco's modified 'Eagle's medium (DMEM; D5030, Sigma-Aldrich, St Louis, MO, USA) containing 10% fetal bovine serum (FBS; F2442; Sigma-Aldrich, St Louis, MO, USA), 100 U/mL Penicillin (Invitrogen, USA), and 100 μg/mL Streptomycin (Invitrogen, USA). To induce osteogenic differentiation, BM-MSCs were divided into Control and OIM groups, and BM-MSCs in OIM groups were treated by a Human Mesenchymal Stem Cell (hMSC) Osteogenic Differentiation Medium Bullet Kit (Lonza, Switzerland) as previously reported.^21^

The plasmids of pcDNA-IRF4, miR-636-mimics, miR-636-inhibitors, si-IRF4-1, and si-IRF4-2 were designed by GeneChem (Shanghai, China) (Supplementary Table 1).

### Alkaline phosphatase (ALP) staining

BM-MSCs were fixed by 4% paraformaldehyde, and the treated cells were cultured for 30 min as per the protocol of an ALP staining kit (Sigma-Aldrich; Merck KGaA). A microscope was used for visualization and a SensoLyte® pNPP Alkaline Phosphatase Assay Kit (Anaspec, USA) was utilized for the quantification of ALP activity.

### Alizarin red staining (ARS)

For ARS staining, BM-MSCs were mixed with Alizarin Red S (Sigma) for 8 min, and the images were captured by a microscope. To determine the level of mineralization, 10% (w/v) cetylpyridinium chloride was used to elute bone nodules, and the absorbance was measured at 562 nm.

### Chromatin immunoprecipitation (ChIP)

To investigate whether IRF4 binds to the promoter region of miR-636, an Auto iDeal ChIP-qPCR Kit (Diagenode, USA) was used in this experiment. In brief, formaldehyde (Sigma-Aldrich, USA) was used to crosslink BM-MSCs, and chromatin was fragmented into 100–300-bp pieces by the sonication method before immunoprecipitation was performed on the remaining DNA fragments using the IRF4 (ab124691, Abcam) or IgG (ab181236, Abcam) Antibody. miR-636 abundance was determined using q-PCR.

### Dual-luciferase reporter assay

To determine the relationship between miR-636 and IRF4 or DOCK9, a luciferase reporter assay was carried out. In brief, IRF4 and control vectors were co-transfected with miR-636-MUT or miR-636-WT into BM-MSCs, respectively. In addition, miR-636 mimics and NC-mimics were co-transfected with DOCK9-MUT or DOCK9-WT into BM-MSCs, respectively, and the luciferase activity of miR-636 and DOCK9 was measured using the Dual-Luciferase Reporter Assay System (Promega, Madison, United States).

### RNA immunoprecipitation assay (RIP)

To determine the correlation between miR-636 and DOCK9, an EZ-Magna RIP kit (Millipore, Billerica, MA, USA) was used in the RIP experiment. In brief, BM-MSCs were treated with RIP buffer, and the cells were then cultured with anti-Ago2- or anti-IgG-conjugated magnetic beads. DOCK9 enrichment was determined by qRT-PCR after extraction of co-precipitated RNAs.

### Western blotting

To detect the expression of target proteins in BM-MSCs, SDS-PAGE was used to separate protein lysates, and the treated samples were transferred onto PVDF membranes, which were then blocked by 5% skimmed milk powder for 1h, and subsequently incubated overnight at 4°C with primary antibodies purchased from Abcam, including OCN (ab198228), OPN (ab214050), Runx2 (ab236639),CollA1 (ab34710), IRF4 (ab133590), DOCK9 (ab204421), GRIK2 (ab247969), NEBL (ab229312), NAV3 (ab201920), C20orf197 (sc-85330, Santa Cruz), and PAPOLA (ab72492). Then, the PVDF membranes were incubated for 1h with the secondary antibody. The protein bands were observed using an Electrochemiluminescence (ECL) kit.

### qRT-PCR

To examine the expression of target genes, NanoDrop 2000 (Thermo, USA) was first utilized to quantify the concentration of RNA extracted from BM-MSCs. A PrimeScript RT Master Mix (Takara, Japan) was then used for reverse transcription, and an SYBR PrimeScript miRNA RT-PCR Kit (Takara, Japan) and SYBR Premix Ex Taq were used to conduct qRT-PCR. U6 acted as the miRNA internal control, and GAPDH acted as the mRNA internal control. All primer sequences are shown in Supplementary Table 2.

### Statistical analysis

In this paper, GraphPad Prism and SPSS 22.0 software was used for data analysis, and the results were shown as mean ± Standard Deviation (SD). *t*-test or analysis of variance (ANOVA) was used to analyze the differences among groups. *p* < 0.05 indicated a significant difference.

## Results

### IRF4 was lowly expressed during osteogenic differentiation

**Fig. 1:**
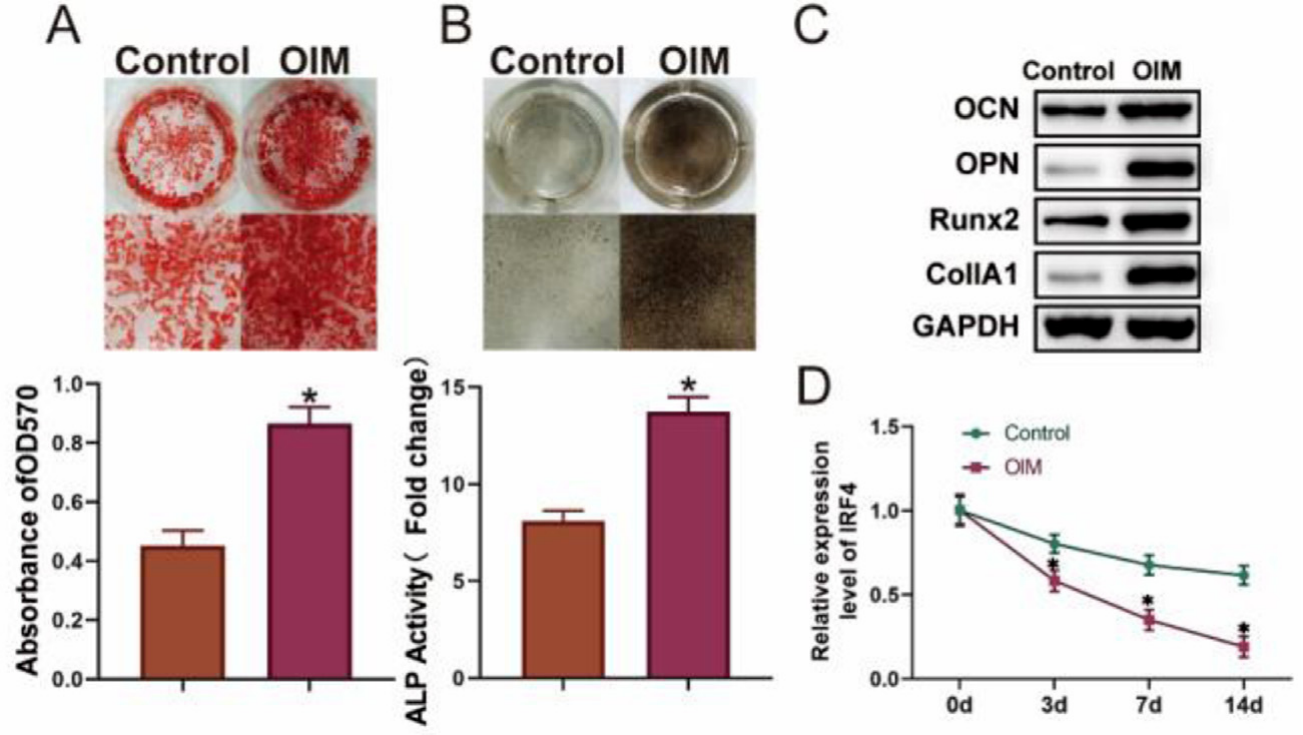
IRF4 is lowly expressed during osteogenic differentiation. ARS (A) and ALP (B) staining assays were applied to evaluate osteogenic differentiation. Western blotting was performed to detect the biomarkers of osteogenic differentiation (C), and q-PCR assay was used to examine IRF4 expression (D). *p < 0.05. Data represent at least three independent sets of experiments.

To verify whether osteogenic differentiation of BM-MSCs was successfully induced, ARS and ALP staining was performed, and it was found that the level of osteogenic differentiation in the OIM group was much higher than that in the Control group, with increased matrix mineralization levels and ALP activity (Fig. 1 A and B). For further investigation, the markers of osteogenic differentiation were examined, and it was found that OCN, OPN, Runx2, and CollA1 in the OIM group were significantly upregulated (Fig. 1C). Next, the expression of IRF4 was monitored in the Control and OIM groups at different time points of osteogenic differentiation (day 0 to day 14), and it was found that IRF4 was lowly expressed during osteogenic differentiation in a time-dependent manner (Fig. 1D). In summary, these results indicated that IRF4 was downregulated during osteogenic differentiation of BM-MSCs.

### The osteogenic differentiation of BM-MSCs was inhibited by IRF4 overexpression

**Fig. 2:**
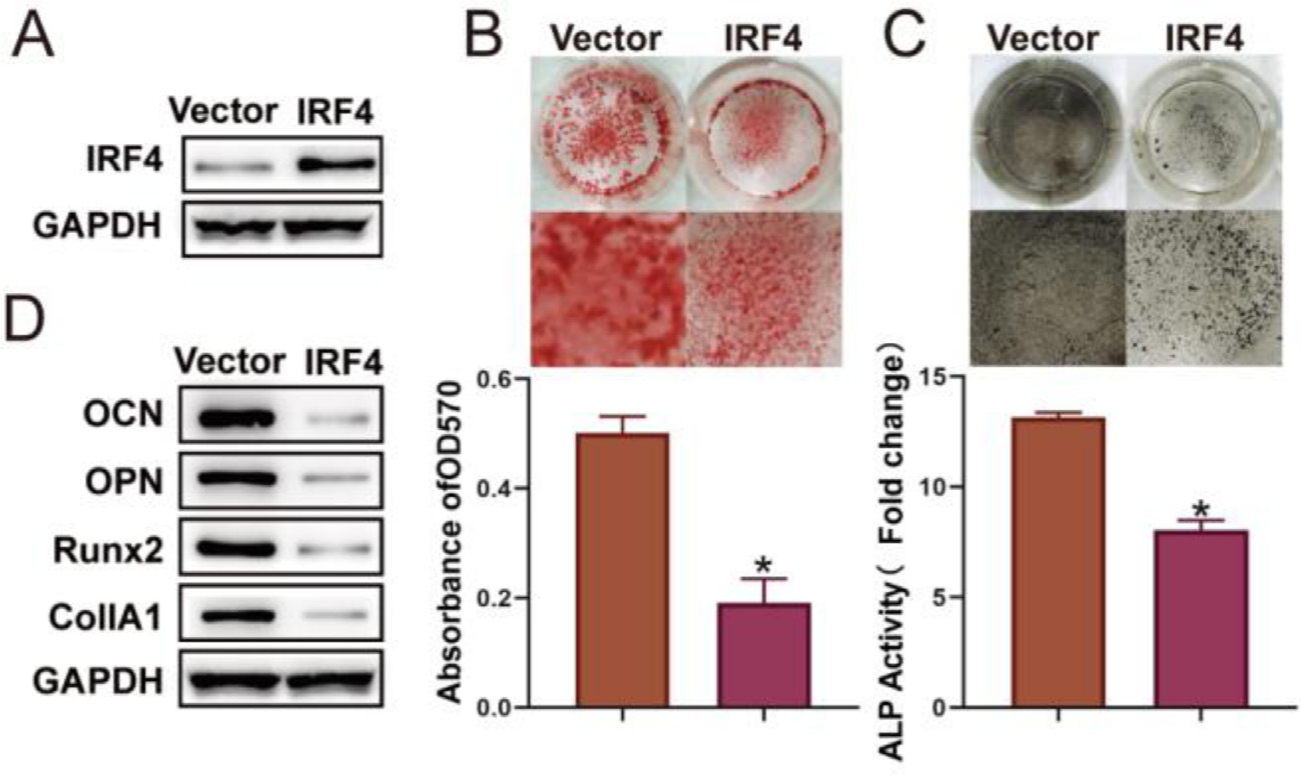
Osteogenic differentiation of BM-MSCs is inhibited by IRF4 overexpression. BM-MSCs with IRF4 overexpression were constructed, and Western blotting was used to examine the transfection efficacy (A). ARS (B) and ALP (C) staining assays were performed for the assessment of osteogenic differentiation. Western blotting was used to detect osteogenic differentiation biomarkers (D). *p < 0.05. Data represent at least three independent sets of experiments.

In order to further investigate the effect of IRF4 on the osteogenic differentiation of BM-MSCs, BM-MSCs with IRF4 overexpression were constructed (Fig. 2A). ARS and ALP staining results showed that the matrix mineralization level and ALP activity were considerably decreased in BM-MSCs transfected with IRF-overexpression plasmids (Fig. 2 B and C). In addition, IRF4 overexpression resulted in reduced expression of markers (OCN, OPN, Runx2 and CollA1) of osteogenic differentiation (Fig. 2D). These findings confirmed that IRF4 overexpression inhibited the osteogenic differentiation of BM-MSCs.

### miR-636 was activated by transcription factor IRF4 in BM-MSCs

**Fig. 3:**
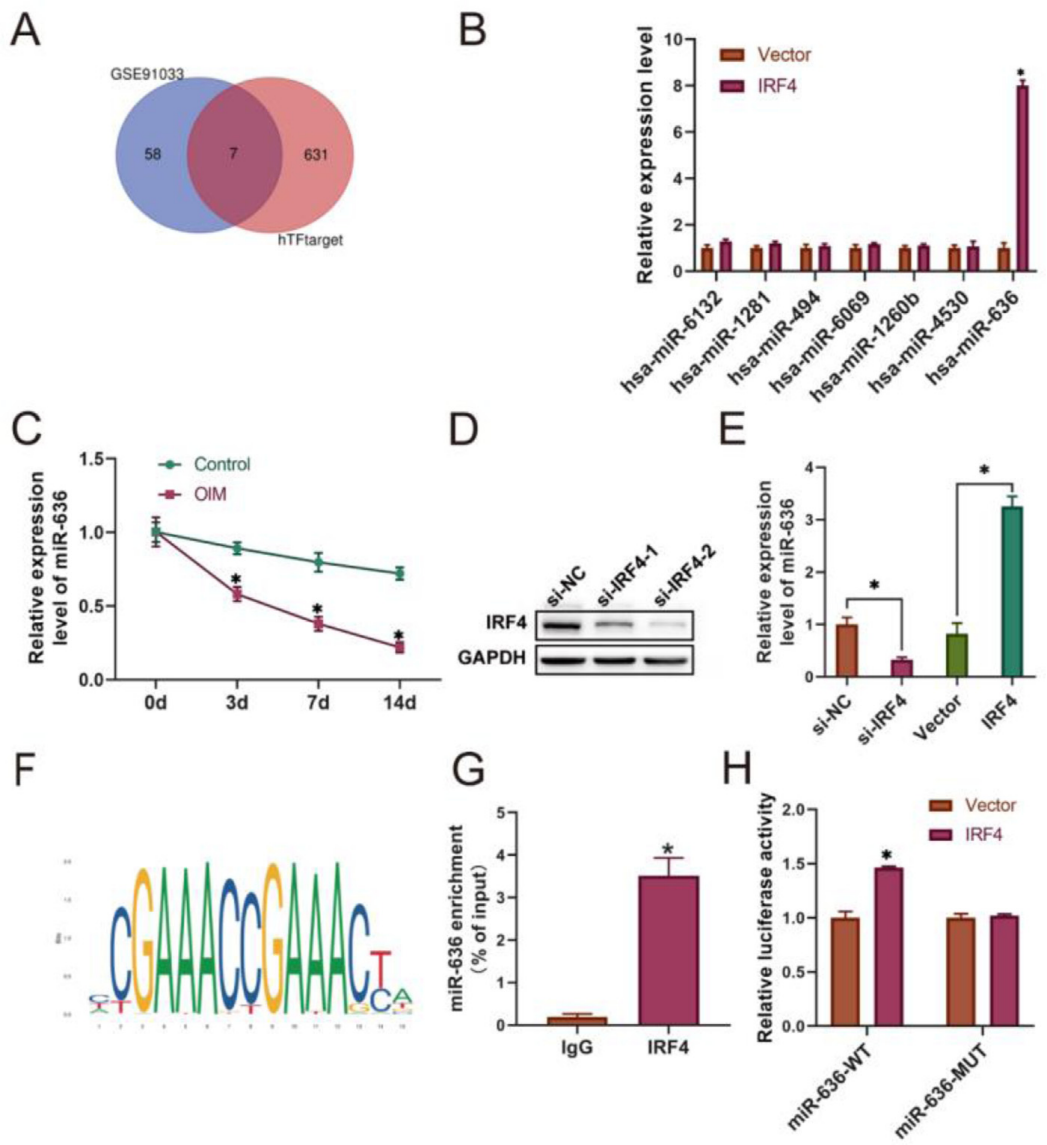
IRF4 activates miR-636 in BM-MSCs. The hTFtarget database and GSE91033 dataset were used to identify the target genes of IRF4 (A), and q-PCR assay was utilized to detect the expression of intersected genes (B-C). IRF4-depletion and IRF4-overexpression BM-MSCs were constructed, and Western blotting and q-PCR were respectively used to determine the transfection efficacy (D) and miR-636 expression (E). The JASPAR database indicated the binding site (F), and the CHIP (G) and Dual Luciferase Reporter (H) assays confirmed the relationship between IRF4 and miR-636. *p < 0.05. Data represent at least three independent sets of experiments.

To investigate the underlying mechanism of IRF4 overexpression inhibiting osteogenic differentiation of BM-MSCs, an intersected network based on the hTFtarget database and GSE91033 was established, and hsa-miR-6132, hsa-miR-1281, hsa-miR-494, hsa-miR-6069, hsa-miR-1260b, hsa-miR-636, and hsa-miR-4530 were identified as potential targets (Fig. 3A). Among them, miR-636 was significantly upregulated in IRF4-overexpression BM-MSCs (Fig. 3B), and miR-636 expression was suppressed after osteogenic differentiation of BM-MSCs over time (Fig. 3C). To further explore the correlation between IRF4 and miR-636, IRF4-depletion and IRF4-overexpression BM-MSCs were constructed, respectively (Fig. 3D). It was noticed that miR-636 was significantly downregulated by IRF4 depletion, which was reversed by IRF4 overexpression (Fig. 3E). Furthermore, the JASPAR database indicated a binding site of IRF4 in the promoter region of miR-636 (Fig. 3F), and the CHIP assay showed that the IRF4 antibody was enriched in miR-636 expression (Fig. 3G). In addition, the Dual-Luciferase Reporter Assay demonstrated that IRF4 overexpression obviously enhanced the luciferase activity of wild-type miR-636, while IRF4 overexpression did not affect the miR-636-MUT group (Fig. 3H). Collectively, these findings revealed that IRF4 could activate miR-636 in BM-MSCs through binding to its promoter region.

### MiR-636 bound to the 3′-UTR of DOCK9

**Fig. 4:**
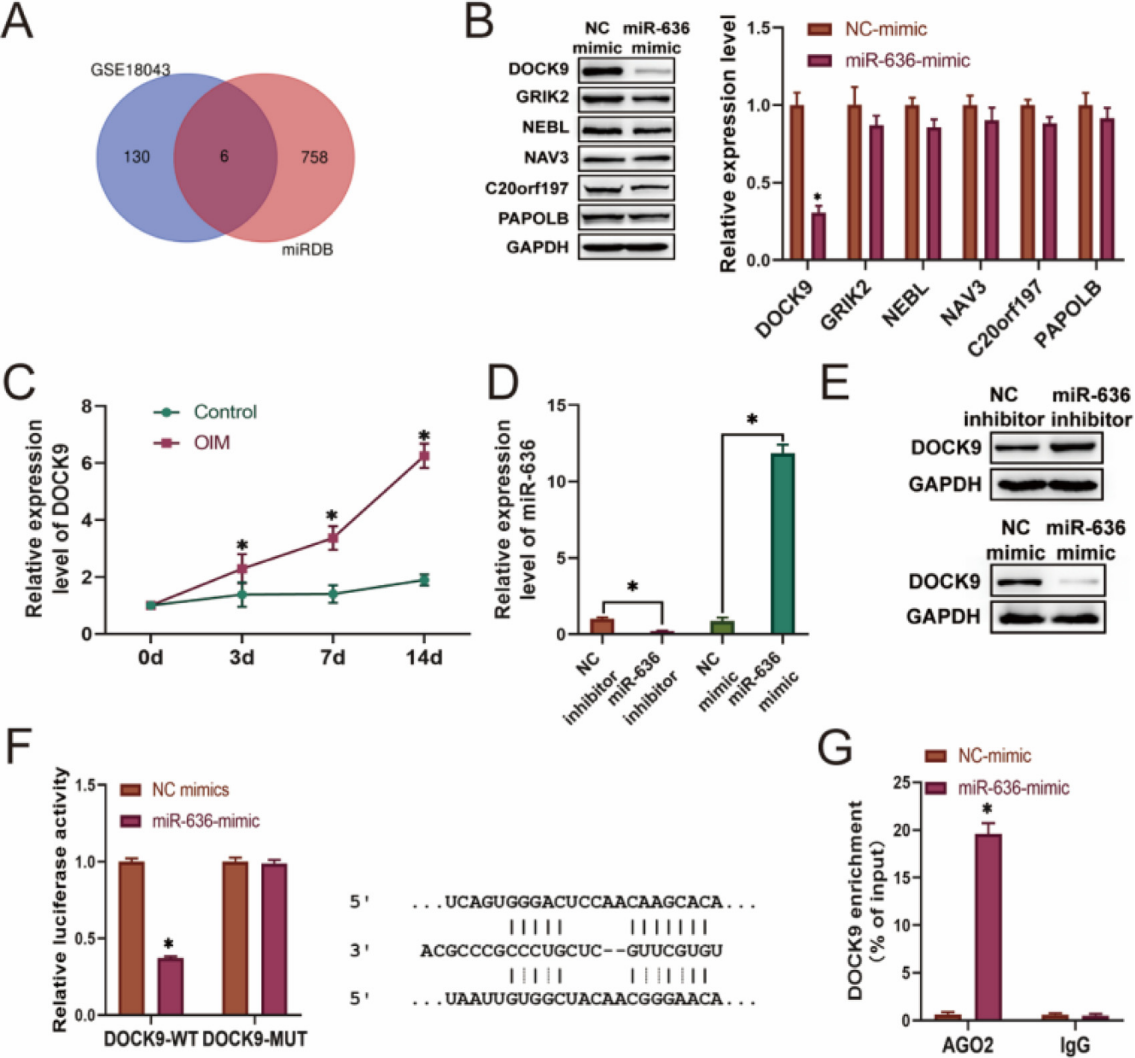
miR-636 binds to the 3′-UTR of DOCK9. The miRDB database and GSE18043 dataset were used to identify the target genes of miR-636 (A), and Western blotting and q-PCR were performed to detect the expression of intersected genes (B). q-PCR results showed the expression of DOCK9 in BM-MSCs (C). BM-MSCs with overexpression or reduced expression of miR-636 were constructed, and q-PCR (D) and Western blotting (E) were respectively utilized to examine the transfection efficacy and DOCK9 expression. Dual Luciferase Reporter (F) and RIP (G) assays were applied to elaborate the relationship between miR-636 and DOCK9. *p < 0.05. Data represent at least three independent sets of experiments.

Subsequently, miRDB was used to predict the target genes of miR-636, and the results were intersected with differentially upregulated genes identified based on the GSE18043 dataset using BM-MSCs that already completed osteogenic differentiation. Six differentially expressed genes were indicated, including DOCK9, GRIK2, NEBL, NAV3, C20orf197, and PAPOLB (Fig. 4A). Among them, DOCK9 was significantly inhibited by miR-636 overexpression (Fig. 4B) and was highly expressed in BM-MSCs over time during osteogenic differentiation (Fig. 4C). To confirm the association between miR-636 and DOCK9, BM-MSCs were transfected with miR-636 inhibitors or miR-636 mimics, respectively (Fig. 4D). Of note, miR-636 silencing increased DOCK9 expression, whereas it was suppressed by miR-636 overexpression (Fig. 4E). In addition, miR-636 mimics could efficiently weaken the luciferase activity of wild-type DOCK9, while it did not affect the DOCK9-MUT group (Fig. 4F). The correlation between DOCK9 and miR-636 was further elaborated by the RIP assay, which showed that miR-636 mimics could enrich DOCK9 mRNA in the AGO2 complex (Fig. 4G). In summary, it was validated that miR-636 could target DOCK9 during osteogenic differentiation of BM-MSCs.

### IRF4 overexpression inhibited the osteogenic differentiation of BM-MSCs via miR-636/DOCK9

**Fig. 5:**
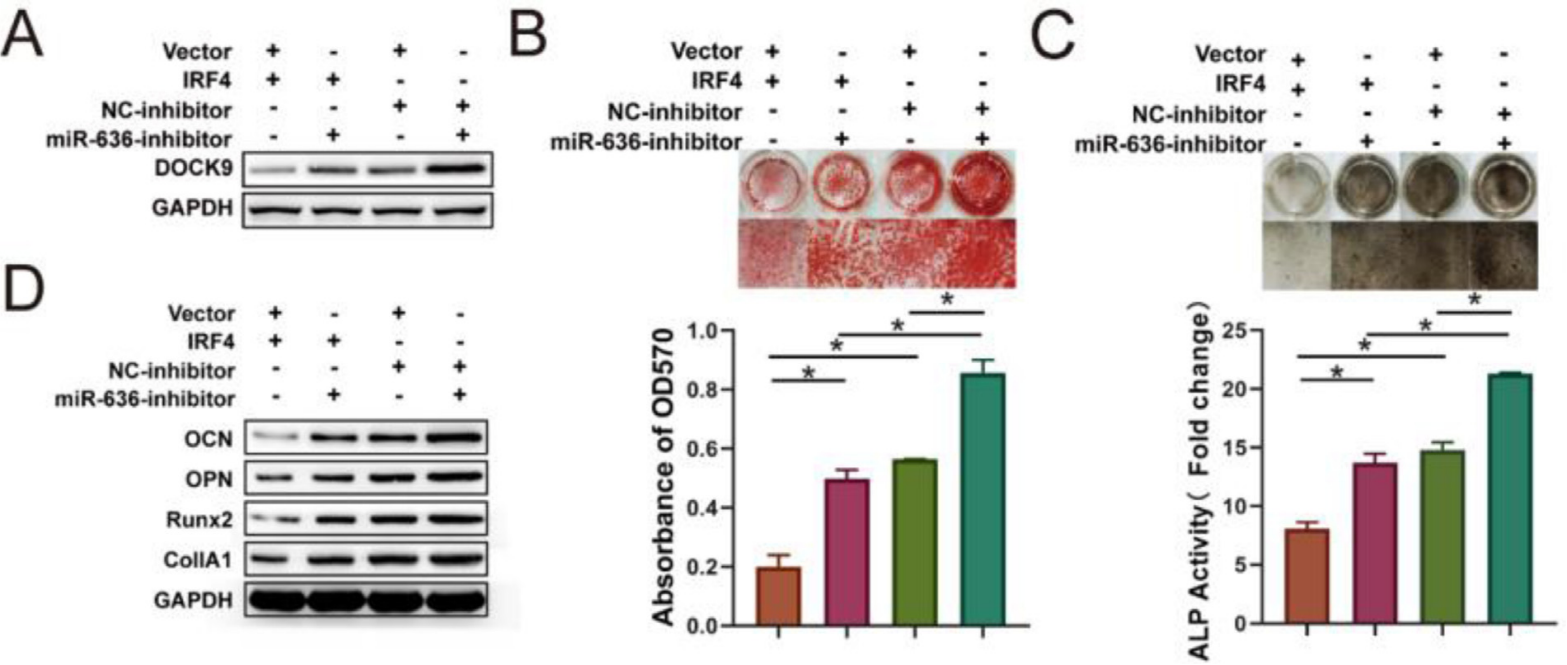
IRF4 overexpression inhibits osteogenic differentiation of BM-MSCs via miR-636/DOCK9. (A) Western blotting was performed to detect DOCK9 expression. ARS (B) and ALP (C) staining assays were performed to determine osteogenic differentiation. Osteogenic differentiation biomarkers were determined by Western blotting (D). *p < 0.05. Data represent at least three independent sets of experiments.

To investigate whether IRF4 overexpression inhibits the osteogenic differentiation of BM-MSCs via the miR-636/DOCK9 axis, BM-MSCs were co-transfected with IRF4 overexpression plasmids and miR-636 inhibitors, and the results showed that IRF4 overexpression could decrease DOCK9 expression. In addition, suppressed DOCK9 expression was rescued by miR-636 inhibition (Fig. 5A). ARS and ALP staining displayed that IRF4 overexpression decreased the matrix mineralization level and ALP activity, but these effects were alleviated by miR-636 silencing (Fig. 5B and C). In addition, depletion of miR-636 played an indispensable role in rescuing the reduction of osteogenic differentiation markers induced by IRF4 overexpression, including OCN, OPN, Runx2, and CollA1 (Fig. 5D). Taken together, these results validated that IRF4 overexpression could inhibit the osteogenic differentiation of BM-MSCs through the miR-636/DOCK9 axis.

## Discussion

Osteoblasts play an indispensable role in bone formation, and BM-MSCs have been considered as progenitors of osteoblasts. The biological process of osteogenic differentiation is directly correlated with the onset and progression of osteoporosis. Recently, the osteogenic differentiation of BM-MSCs has attracted 'scholars' eyes. For instance, Molagoda et al. demonstrated that Fisetin could accelerate the osteoblast differentiation through activating the phosphorylation of GSK-3β at Ser9, thereby ameliorating osteoporosis.^22^ Chen et al. reported a positive association between Scara3 and osteogenic differentiation of BM-MSCs.^23^ Nevertheless, biomarkers of osteogenic differentiation are still limited.

In this study, transcription factor IRF4 was downregulated during osteogenic differentiation of BM-MSCs, which was reversed overtime during osteogenic differentiation. In addition, it was revealed that IRF4 overexpression suppressed osteogenic differentiation via reducing matrix mineralization levels, ALP activity, and expressions of OCN, OPN, Runx2, and CollA1. Consistent with the findings of this paper, previous studies have shown the effects of various transcription factors on osteogenic differentiation. For example, Ye et al. highlighted that IRF2 could bind to the promoter region of lncRNA HHAS1 to drive its activation, thereby promoting the osteogenic differentiation of BM-MSCs via mediating the miR-204-5p/RUNX2 axis.^24^ Xu et al. found that ZEB1 could negatively regulate osteogenic differentiation of BM-MSCs via the Wnt/β-Catenin signaling pathway.^25^ In addition, Wu and colleagues indicated that NFIX-activated HMGA1 promotes osteogenic differentiation via stimulating the Wnt signaling pathway.^26^ Herein, miR-636 was identified as the downstream miRNA of IRF4, and IRF4 could activate miR-636 expression through binding to its promoter region.

MicroRNAs have been validated to be involved in the regulation of osteogenic differentiation. For example, it has been revealed that miR-100-5p derived from exosomes inhibited the BMPR2/Smad1/5/9 signaling pathway to prevent the osteogenesis of BMSCs.^27^ Wei et al. revealed that exosomal miR-424-5p derived from bone marrow stem cells prevented osteogenesis through targeting WIF1.^28^ In this study, it was found that miR-636 inhibition could accelerate the osteogenic differentiation of BM-MSCs. In addition, based on the informatics analysis and *in vitro* experiments such as RIP and Dual-Luciferase Reporter assays, DOCK9 was confirmed as a target of miR-636. Few studies have reported the role of DOCK9 in osteoporosis. Olivia L Sabik et al. identified DOCK9 as one of the core modules for bone mineral density through integrating a Co-expression Network and GWAS Data.^29^ In the present study, DOCK9 overexpression promoted the osteogenic differentiation of BM-MSCs, suggesting that it might be involved in bone formation. More importantly, miR-636 depletion played an indispensable role in recovering the impairment of osteogenic differentiation induced by IRF4 overexpression in BM-MSCs.

The present study found that IRF4 was downregulated in osteogenic differentiation of BM-MSCs, and IRF4 overexpression inhibited osteogenic differentiation. IRF4 could activate miR-636 via binding to its promoter region, and DOCK9 was identified as the target of miR-636 in BM-MSCs. In addition, it was disclosed that the inhibited osteogenic differentiation of BM-MSCs induced by IRF4 overexpression was rescued by miR-636 inhibition. In summary, IRF4/miR-636/DOCK9 axis was a promising target for the treatment of osteoporosis.

## Authors' contributions

Xuepu Zhang, Yue Zhang and Haosen Zhao conceived the study and designed the experiments. Limin Yang, Yuexin Wu and Xiaohu Ma contributed to the data collection. Gang Tong and Zhaoliang Ban performed the data analysis and interpreted the results. Xuepu Zhang and Yue Zhang wrote the manuscript. Haosen Zhao contributed to the critical revision of the article. All authors read and approved the final manuscript.

## Conflicts of interest

The authors declare no conflicts of interest.

## Appendix Supplementary materials

Image, application 1
