# Supplementary material for: IRF4 suppresses osteogenic differentiation of BM-MSCs by transcriptionally activating miR-636/DOCK9 axis
Source: Clinics (Sao Paulo). 2022 Apr 6;77:100019. doi: 10.1016/j.clinsp.2022.100019 (PMC8989710; doi:10.1016/j.clinsp.2022.100019)
Supplement: Supplementary file 1 [file mmc1.pdf]

CLINICS-2021-3580 – Supplementary Material

Supplementary Table 1 Sequences of siRNA against specific targets.

si- IRF4-1	5’-3'	TTGAACAAGAGCAATGACTTTGA
si- IRF4-2	5’-3’	CGGCACGCGGGGCATGAACCTGG

Supplementary Table 2 Sequences of PCR primers used in this study.

IRF4	Forward (5'-3'3')	GCTGATCGACCAGATCGACAG
	Reverse (5’-3’)	CGGTTGTAGTCCTGCTTGC
miR-636	Forward (5’-3')	AGTGTGCTTGCTCGTC
	Reverse (5’-3’)	GTCCAGTTTTTTTTTTTTTTTGCG
DOCK9	Forward (5’-3’)	TGTCATCGTCCAGAAGAAGACT
	Reverse (5’-3’)	TCTCAGGATGGCCGTCTGAAA
GRIK2	Forward (5’-3’)	TTCAGGCGCACCGTTAAACT
	Reverse (5’-3’)	GCTCCCATTGGGCCAGATT
NEBL	Forward (5’-3’)	AGAGGCTTTACTCCCGTCGT
	Reverse (5’-3’)	ACCCCTTTATAGGCAGCATCG
NAV3	Forward (5’-3’)	AGCCTGTGCATACTGCTCTTC
	Reverse (5’-3’)	TGATTTTAACGCAAGCTGACAAG
C20orf197	Forward (5’-3’)	CAGCCCTTATTGGCAGGCA
	Reverse (5’-3’)	AGTGTGGGATGGACTATGTTCA
PAPOLB	Forward (5’-3’)	ATGATGCCGTTTCCGGTGAC
	Reverse (5'-3'3')	GCTAGACTGATAGGCGAGGAGA
hsa-miR-6132	Forward (5’-3')	GCAGGGCTGGGGAT
	Reverse (5’-3’)	TCCAGTTTTTTTTTTTTTTTGCAATC
hsa-miR-1281	Forward (5’-3')	AGTCGCCTCCTCCTCT
	Reverse (5’-3’)	GTCCAGTTTTTTTTTTTTTTTGGGA
hsa-miR-494	Forward (5’-3’)	GGTTGTCCGTGTTGTCTTC
	Reverse (5’-3’)	GGTCCAGTTTTTTTTTTTTTTTAGAGA
hsa-miR-6069	Forward (5’-3’)	GGCTAGGGCCTGCTG
	Reverse (5’-3’)	GTTTTTTTTTTTTTTTGGGGGCA
hsa-miR-	Forward (5’-3’)	GATCCCACCACTGCCA

1260b	Reverse (5’-3’)	GGTCCAGTTTTTTTTTTTTTTTATGG
hsa-miR-4530	Forward (5’-3')	CCAGCAGGACGGGA
	Reverse (5’-3’)	TCCAGTTTTTTTTTTTTTTTCGCT
GAPDH	Forward (5’-3’)	GGAGCGAGATCCCTCCAAAAT
	Reverse (5’-3’)	GGCTGTTGTCATACTTCTCATGG
U6	Forward (5’-3’)	GCGCGTCGTGAAGCGTTC
	Reverse (5’-3')	GTGCAGGGTCCGAGGT
